# Therapeutic use of highly concentrated CO_2_ for wound healing: bathing and non-bathing modalities in a systematic review and meta-analysis

**DOI:** 10.7717/peerj.21189

**Published:** 2026-05-05

**Authors:** Shaodong Chen, Jia Ying Tong, Muhammad Shahzad Aslam

**Affiliations:** 1 School of Traditional Chinese Medicine, Xiamen University Malaysia, Jalan Sunsuria, Bandar Sunsuria, Selangor, Malaysia; 2 Hepatology Unit, Xiamen Hospital of Traditional Chinese Medicine, Xiamen, Fujian, China; 3 Department of Traditional Chinese Medicine, School of Medicine, Xiamen University, Xiamen, Fujian, China; 4 Western Medicine Unit, School of Traditional Chinese Medicine, Xiamen University Malaysia, Sunsuria, Selangor, Malaysia

**Keywords:** Carbon dioxide therapy, Wound healing, Meta-analysis, Chronic wounds, Randomized controlled trial, Pressure ulcer, Diabetic foot ulcer

## Abstract

**Background:**

Wound healing poses a persistent clinical challenge, especially in chronic wounds like diabetic foot ulcers and pressure injuries. Highly concentrated carbon dioxide (CO_2_) therapy has emerged as a non-invasive approach to promote healing, but its effectiveness remains uncertain.

**Objective:**

To evaluate the efficacy of highly concentrated CO_2_ therapy, *via* bathing and non-bathing methods, on wound healing outcomes.

**Methods:**

This systematic review and meta-analysis followed PRISMA guidelines and was registered in PROSPERO (CRD420251035698). Six databases were searched, identifying 10,348 records. Five randomized controlled trials met the inclusion criteria, involving participants with wounds treated using highly concentrated CO_2_ through bathing or non-bathing methods. Two reviewers independently extracted data and assessed risk of bias using the Cochrane RoB 2.0 tool. Meta-analyses were conducted with fixed- or random-effects models, and the certainty of evidence was evaluated using the GRADE approach.

**Results:**

Five trials (*N* = 127 wounds/participants) were included. Meta-analysis of two trials indicated that CO_2_ therapy increased the likelihood of complete ulcer healing (RR = 5.33; 95% CI [0.23–126.05]; *I*^2^ = 81.3%), though the evidence was very uncertain due to heterogeneity and imprecision. Another meta-analysis of two trials found moderate improvement in microvascular perfusion (SMD = 0.61; 95% CI [0.23–0.99]; *I*^2^ = 0%), rated as low certainty. Individual studies reported improvements in skin temperature, VEGF, TNF-α, and wound area reduction.

**Conclusion:**

Highly concentrated CO_2_ therapy shows promise in enhancing wound healing. However, further large-scale, high-quality trials across diverse settings are needed to validate its clinical applicability.

## Introduction

Delayed wound closure increases the risk of infection, amputation, and decreased quality of life, while also putting a strain on healthcare resources due to frequent clinic visits and extended hospital stays. In 2014, 15% of Medicare beneficiaries, approximately 8.2 million individuals, experienced one or more wounds or infections. Among these, surgical infections were the most common, affecting 4.0% of beneficiaries, while diabetic infections accounted for 3.4%. Total expenditures for these conditions ranged from $28.1 billion to $96.8 billion, with the highest costs associated with surgical wounds ($11.7 billion to $38.3 billion) and diabetic foot ulcers ($6.2 billion to $18.7 billion). Outpatient care expenditures were between $9.9 billion and $35.8 billion, compared to $5.0 billion to $24.3 billion for inpatient services (Nussbaum et al., 2018).

Conventional treatment includes debridement, offloading, dressings, compression, and the optimization of nutritional and glycemic management does not fully heal as many as 30 percent of chronic ulcers, highlighting the necessity for innovative additional therapies (Weller et al., 2016). Diabetes mellitus, marked by persistent hyperglycemia, impairs wound healing in about 25% of patients, frequently leading to lower-limb amputation and substantial economic and psychosocial burdens (Burgess et al., 2021). Standard care for diabetic neuropathic foot ulcers achieves only a 31% healing rate at 20 weeks (Margolis, Kantor & Berlin, 1999), highlighting a clear need for more effective intervention.

One of the primary factors delaying wound healing in diabetic patients is wound hypoxia, which contributes to the development of chronic, non-healing wounds (Guan et al., 2021). Diabetic wound healing involves three overlapping stages: inflammation, proliferation, and remodeling (Wallace, Basehore & Zito, 2023). Impaired vasculature in chronic diabetes hampers local blood circulation, resulting in ischemic wounds (Dasari et al., 2021). Prolonged oxygen deprivation negatively affects angiogenesis and tissue perfusion, extends the inflammatory phase, and increases the production of reactive oxygen species (ROS), ultimately exacerbating tissue damage (Wernick, Nahirniak & Stawicki, 2023). Additionally, immune dysfunction in diabetic patients delays the cascade of immune responses at the wound site (Berbudi et al., 2019). Neutrophils, a type of polymorphonuclear leukocyte, play essential roles in pathogen clearance, inflammation control, and tissue regeneration by promoting angiogenesis and extracellular matrix (ECM) remodeling (Yang et al., 2025). However, decreased neutrophil function in diabetic individuals can compromise phagocytosis and tissue repair, leading to wound infection and chronicity.

Carbon dioxide (CO_2_), a naturally exhaled gas, has recently gained attention as a promising treatment for various wounds, particularly diabetic and infected wounds. CO_2_ therapy enhances local blood flow and oxygenation, reduces inflammation, and accelerates wound healing (Prazeres, Lima & Ribeiro, 2025). It is typically administered either by bathing the wound area in CO_2_-enriched water at controlled temperatures (37 °C) or *via* transcutaneous gaseous application (Finžgar et al., 2021). Recent technological advancements have enabled the use of highly concentrated CO_2_ in tablet form, making at-home application feasible. In Japan, these tablets are widely available, low-cost (approximately USD 1 per day), and increasingly used for wound care (Hihara et al., 2024). The positive outcomes associated with high-CO_2_ exposure support its potential role as an effective adjunct to standard treatment.

In this context, highly concentrated CO_2_ delivered through both bathing and non-bathing methods has emerged as a potential therapy because of its reported benefits on microvascular perfusion, angiogenesis, and antimicrobial activity. However, individual CO_2_ therapy trials often report only modest improvements in ulcer healing and are generally underpowered to detect true effects. This limitation warrants a systematic review and meta-analysis to generate a pooled estimate, assess variability, and determine whether CO_2_ interventions consistently produce statistically significant improvements in microvascular perfusion or complete ulcer healing. Therefore, our systematic review and meta-analysis aim to evaluate whether CO_2_-based treatments can significantly improve upon the suboptimal outcomes associated with conventional wound care.

Despite increasing interest in carbon dioxide-based wound therapies, the existing evidence remains inadequate for clinical decision-making. Current studies are fragmented across different delivery modalities, including transcutaneous gas application, injections, and water-based systems, and employ heterogeneous comparator designs, ranging from routine care to placebo-controlled approaches. In addition, the components of standard wound care are often inconsistently defined or insufficiently reported, limiting meaningful comparison across studies. Clinically relevant outcomes, particularly complete wound healing, are also reported inconsistently and are frequently underpowered. Furthermore, prior literature has largely remained descriptive, with limited quantitative synthesis and no comprehensive assessment of certainty of evidence. The present systematic review and meta-analysis addresses these gaps by providing a structured synthesis of randomized evidence, incorporating quantitative pooling where feasible, risk-of-bias evaluation, and GRADE-based certainty assessment.

## Methods

### Protocol and registration

This systematic review and meta-analysis were conducted in accordance with the Preferred Reporting Items for Systematic Reviews and Meta-Analyses (PRISMA) guidelines. The protocol was registered in the PROSPERO database under registration number CRD420251035698. The review adheres to PRISMA 2020; the completed checklist is provided in Appendix S1.

### Information sources and search strategy

A total of 10,348 records were retrieved from major electronic databases, including PubMed, PubMed Central (PMC), Web of Science (WOS), Scopus, ClinicalTrials.gov, and Google Scholar. A comprehensive search strategy was developed using a combination of keywords, Medical Subject Headings (MeSH), and free-text terms related to carbon dioxide therapy and wound healing. Boolean operators (AND, OR) were employed to appropriately broaden or narrow the scope of the search. A summary of the search strategies used across each database is presented in Appendix S2.

### Selection process

**Figure 1:**
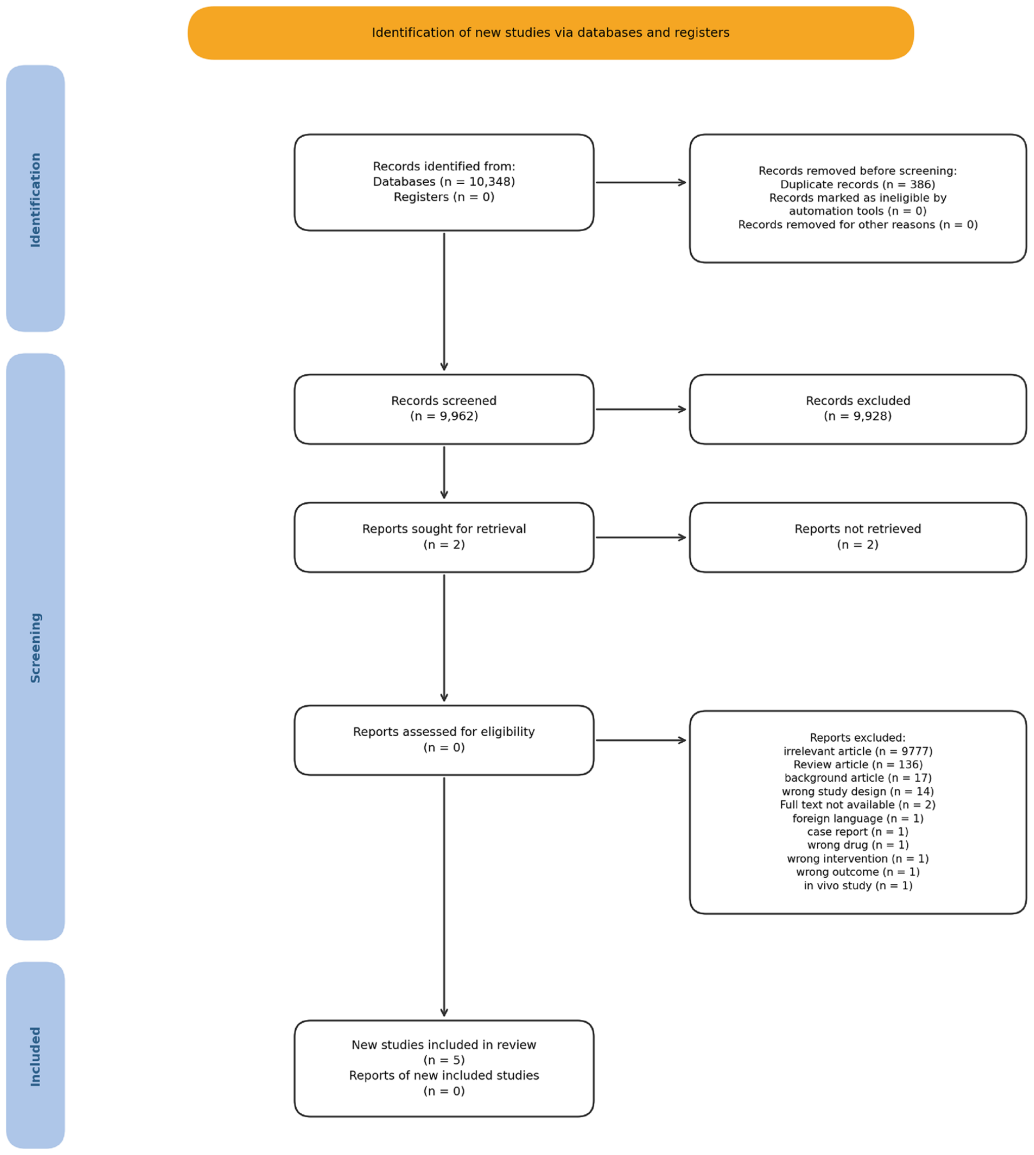
PRISMA 2020 flow diagram. The identification, screening, and inclusion of studies in the systematic review. A total of 10,348 records were identified from databases, and 386 duplicates were removed before screening. Of the 9,962 records screened, 9,928 were excluded for reasons including irrelevant content (*n* = 9,777), review articles (*n* = 136), background articles (*n* = 17), wrong study design (*n* = 14), full text not available (*n* = 2), foreign language (*n* = 1), case report (*n* = 1), wrong drug (*n* = 1), wrong intervention (*n* = 1), wrong outcome (*n* = 1), and *in vivo* study (*n* = 1). Two reports were sought for retrieval but could not be obtained. Ultimately, five new studies were included in the review.

Two reviewers (MS Aslam and JY Tong) independently screened the titles and abstracts of all retrieved records using Rayyan software (Ouzzani et al., 2016). The selection process was double-blinded, meaning each reviewer was unaware of the other’s decisions. Any discrepancies in search terms, databases, or results screening were resolved through discussion. If consensus could not be reached, the issue was referred to Chen Shaodong for adjudication. Literature screening was conducted on 19 April 2025, when all identified records were uploaded and screened in Rayyan, and data extraction was carried out during June 2025. A total of 386 duplicate entries were automatically removed by the software, resulting in 9,962 records being screened and 9,928 records excluded. Two reports were sought for full-text retrieval, but could not be obtained. The reasons for exclusion were as follows: 9,777 irrelevant articles, 136 review articles, 17 background articles, 14 with the wrong study design, two full texts not available, one non-English article, one case report, one wrong drug, one wrong intervention, one wrong outcome, and one *in vivo* study. In total, five studies were included in the systematic review and meta-analysis (Finžgar et al., 2021; Ban Frangež et al., 2021; Brandi et al., 2010; Abdulhamza & Mohammed, 2024; Macura et al., 2020). Discrepancies in study inclusion were resolved through discussion and mutual consensus. The study selection process will be documented in a PRISMA 2020 flow diagram, which will be provided in as Fig. 1.

#### Duplicate removal strategy

To ensure methodological rigor and eliminate redundant records retrieved from multiple databases, a standardized de-duplication process was performed using the Systematic Auto Resolver within Rayyan QCRI (Ouzzani et al., 2016). The auto-resolution tool was configured with a minimum similarity threshold of 80%, which assessed textual resemblance between bibliographic entries while allowing for minor variations in metadata formatting. Duplicates were primarily identified based on exact matches in the title and year fields. Additional settings, such as Text Normalization and Articles Customization Protection, were enabled to improve detection sensitivity and preserve user annotations. When multiple versions of the same article were detected, Rayyan’s algorithm retained the version with the most complete metadata. The deduplicated dataset was manually reviewed to ensure accuracy. Rayyan notes that thresholds below 95% may increase false positives; thus, manual verification of resolved duplicates was conducted to minimize data loss or unintended exclusions.

### Eligibility criteria

Only studies published in English were included. Eligible studies involved human participants of any age, sex, or ethnicity with either acute or chronic wounds, including surgical wounds, pressure ulcers (bedsores), diabetic foot ulcers, venous leg ulcers, burn wounds, and traumatic skin injuries. Participants could be from inpatient, outpatient, or community healthcare settings. Included studies assessed wound healing outcomes using highly concentrated carbon dioxide (CO_2_) therapy delivered *via* bathing or non-bathing methods such as transcutaneous gas application, injections, transdermal patches, inhalation, or CO_2_-infused hydrogels. Studies were excluded if they involved participants without wounds, addressed conditions unrelated to wound healing, or were animal or *in vitro* (cell culture) studies. Additionally, studies focusing solely on cosmetic or dermatological applications of CO_2_ without reporting wound healing outcomes were excluded, as were those in which wound healing was not assessed as a primary or secondary outcome.

### Data collection process and data items extracted

Two reviewers (MS Aslam and JY Tong) independently extracted data using a standardized data extraction form. Any discrepancies were resolved through discussion prior to finalizing the dataset. Data extracted from each study included author information, year of publication, country of origin, study design, participant demographics such as sample size, intervention, and comparator details including type and duration of CO_2_ therapy, outcomes assessed, and key surrogate effects. The extracted data were compiled into a summary table presented in Table 1.

### Risk of bias and quality assessment

The risk of bias for randomized controlled trials was independently assessed by two reviewers (MS Aslam and JY Tong) using the Cochrane Risk of Bias 2.0 (RoB 2.0) tool, which evaluates bias across five domains: the randomization process, deviations from intended interventions, missing outcome data, measurement of outcomes, and selection of the reported result. Each domain was rated as having low risk, some concerns, or high risk of bias. A structured Excel template from the ROBVIS website was used to facilitate the assessment process (McGuinness & Higgins, 2021), and visual risk-of-bias plots were generated using the ROBVIS tool to enhance clarity and transparency (Haddaway & Grainger, 2025). Discrepancies between reviewers were resolved through discussion and consensus.

**Table 1:** Characteristics of the studies included in the systematic review (*n* = 5).

**Study (country; design & modality)**	**Participants (CO_2_/control)**	**Treatment schedule**	**Primary healing outcome**	**Key surrogate/physiological effects**
**Frangež 2021** (Slovenia; double-blind, transcutaneous dry-gas wrap) (Ban Frangež et al., 2021)	30/30 patients	45-min wraps, 5 × week for 4 weeks	**Not reported**	Big-toe temperature rose to 30.7 ± 1.6 °C from 26.3 ± 2.9 °C (*p* < 0.001); vibration-perception and monofilament error scores both roughly halved (*p* < 0.001).
**Finžgar 2021** (Slovenia; double-blind, transcutaneous dry-gas wrap) (Finžgar et al., 2021)	21/21 subjects	Same wrap protocol as above	**Not reported**	NO-mediated oscillatory power increased from 0.113 ± 0.108 to 0.154 ± 0.101 (*p* = 0.015); peak thermal-hyperaemia response rose to 482 ± 474% of baseline (*p* = 0.036).
**Macura 2020** (Slovenia; double-blind, transcutaneous dry-gas wrap) (Macura et al., 2020)	30 wounds/27 wounds	20 daily sessions over 4 weeks	Complete healing in ** 20/30** wounds *vs*** 0/27**; median area reduction 96% (IQR 99–88) *vs* 25% (41–9) (*P* = .001).	Wound volume fell by 99% *vs* 27% (*P* < .001); Falanga score shifted to grade A in 93% of treated wounds.
**Brandi 2010** (Italy; open-label, sub-cutaneous CO_2_ injections) (Brandi et al., 2010)	35/35 patients	Two injection sessions per week for 6 weeks	Complete healing in ** 25/35** patients *vs*** 18/35**; mean healing time 25 days *vs* 37 days.	TcPO_2_ increased from 21.6 ± 10.6 to 41.2 ± 16.7 mm Hg (*p* < 0.001); resting laser-Doppler flux rose from 11.2 ± 1.7 to 26.1 ± 3.5 PU (*p* = 0.003).
**Abdulhamza 2024** (Iraq; double-blind, CO_2_ water bath) (Abdulhamza & Mohammed, 2024)	95 total; arm counts ** NR**	30-min foot baths, 3 × week for 3 months	Ulcer closure in ** 66%** of CO_2_ group *vs*** 0%** placebo (denominators not reported).	VEGF rose and TNF-α declined significantly in CO_2_ group (exact means not tabulated; both *p* < 0.001).

**Notes.**

Key methodological and outcome details for each trial that evaluated carbon dioxide based interventions in chronic wound care. Columns include:

Study: first author, year, country, trial design, and delivery modality.

Participants: number in intervention and control groups.

Treatment schedule: session duration, frequency, and total treatment period.

Primary healing outcome: complete healing rate or wound area reduction.

Key surrogate or physiological effects: perfusion, biochemical, or sensory endpoints that support the proposed mechanism.

Abbreviations CO_2_ carbon dioxide NO nitric oxide TcPO_2_ transcutaneous partial pressure of oxygen PU perfusion units IQR interquartile range VEGF vascular endothelial growth factor TNF-α tumour necrosis factor alpha NR not reported p two-sided probability value

### Certainty of evidence assessment

We assessed the certainty of evidence for each outcome using the GRADE approach. Evidence profiles were developed based on risk of bias, inconsistency, indirectness, imprecision, and potential publication bias. Two outcomes were evaluated: resting skin perfusion (continuous) and complete ulcer healing (dichotomous). For each, we extracted data from eligible randomised controlled trials and calculated pooled effect estimates using fixed-effect or random-effects models as appropriate. Results for continuous outcomes were reported as standardised mean differences (SMDs) with 95% confidence intervals (CIs); results for dichotomous outcomes were reported as risk ratios (RRs). We rated the certainty of evidence as high, moderate, low, or very low according to GRADE guidance (Schünemann et al., 2013). Downgrading decisions were explicitly documented for each domain. Evidence tables were prepared in accordance with GRADE recommendations.

### Effect measures and data synthesis

For continuous outcomes, mean differences (MD) or standardized mean differences (SMD) with 95% confidence intervals (CIs) were calculated. For dichotomous outcomes, risk ratios (RR) and 95% CIs were reported. Meta-analyses were conducted using a random-effects model when substantial heterogeneity was present, as assessed by the I^2^ statistic and Chi-square test. A fixed-effect model was applied when heterogeneity was minimal. All meta-analyses were performed in R (RStudio) using the meta and metafor packages.

### Outcomes

Primary outcomes focused on wound-closure metrics, including: (1) wound healing rate, defined as the rate at which wound size decreased over time, measured using digital planimetry, rulers, or imaging software; (2) time to complete wound closure, referring to the duration required for full epithelialization, assessed through clinical observation or digital documentation; and (3) wound area reduction, calculated as the change in wound surface area from baseline to follow-up. All primary outcomes were reported as either mean differences (MD) or standardized mean differences (SMD) with 95% confidence intervals (CIs), depending on the measurement scale and outcome type.

Secondary outcomes included surrogate markers of tissue perfusion and angiogenesis, such as transcutaneous oxygen tension (TcPO_2_), laser-Doppler blood flow, thermal imaging, and capillary density assessments. Additionally, biomarkers of microvascular function, including vascular endothelial growth factor (VEGF) and nitric oxide (NO), were considered, along with other physiological parameters predictive of wound healing.

### Statistical analysis and reproducibility

All meta-analyses were performed in R (RStudio) using the meta and metafor packages. The full reproducibility including the Rayyan screening, registered protocol (PROSPERO CRD420251035698), and the R script for all analyses is openly available on the Open Science Framework (OSF; DOI: 10.17605/OSF.IO/F8PGV).

For complete ulcer healing rates, we applied a random-effects model to account for anticipated clinical and methodological variability across trials. Although heterogeneity was high (I^2^ = 81%), the studies addressed the same outcome in broadly comparable patient populations, and pooling was considered appropriate for exploratory purposes. We acknowledge that sparse-event data limit the precision of the pooled estimate. To mitigate this, a continuity correction was applied for studies with zero events, in line with Cochrane Handbook guidance (Altman et al., 2021). Consequently, the pooled effect should be interpreted cautiously, emphasizing the very low certainty of evidence.

### Use of Artificial Intelligence (AI)

ChatGPT 3.o and ChatGPT 4.0 (OpenAI) was used as an assistive tool at predefined points to: (i) generate first-pass drafts of the study-level data-extraction table (study characteristics and outcome values) from included PDFs and plain-language GRADE rationales; and (ii) provide text scaffolds for specific sections (Abstract; Eligibility Criteria; PRISMA selection text/legend; Duplicate-removal description; heterogeneity interpretation; RoB 2 results text/legend; Discussion subsections on microvascular outcomes, biomarkers, healing uncertainty, modality variation, risk of bias, and safety) and code templates for the meta-analyses (complete-healing RR with continuity correction/REML and laser-Doppler SMD with sensitivity analysis). Two reviewers independently verified all extracted values against source articles and finalized all GRADE ratings/rationales; eligibility decisions, RoB 2 judgments, statistical modelling, and the final manuscript text were performed and approved by the authors. Prompts and representative AI outputs are available on OSF (DOI: 10.17605/OSF.IO/F8PGV).

## Results

Five randomized controlled trials, encompassing a total of 127 participants or wounds, have investigated the efficacy of concentrated carbon-dioxide therapy for the treatment of chronic ulcers. In the study conducted by Ban Frangež et al. (2021), thirty patients received CO_2_ wraps, while thirty others were assigned to air wraps. After four weeks, the mean skin temperature of the big toe in the intervention group rose from 26.3 °C (SD 2.9) to 30.7 °C (SD 1.6, *p* < 0.001). Additionally, significant reductions were observed in both vibration-perception and monofilament scores, with both changes achieving statistical significance. Abdulhamza & Mohammed (2024) enrolled ninety-five participants and reported that approximately two-thirds of the ulcers in the CO_2_ therapy group achieved complete healing, whereas no ulcers healed in the control group receiving advanced dressings and antibiotics. Furthermore, levels of vascular endothelial growth factor (VEGF) increased significantly, while tumor necrosis factor-alpha (TNF-α) levels decreased markedly, each with *p* < 0.001, although specific denominators for the groups were not disclosed. Brandi’s study involved thirty-five patients in each treatment arm, comparing subcutaneous CO_2_ injections to standard care. The transcutaneous oxygen tension increased significantly from 21.6 mm Hg (SD 10.6) to 41.2 mm Hg (SD 16.7, *p* < 0.001). Moreover, laser-Doppler flux showed an increase of 14.9 perfusion units (*p* = 0.003), with wound healing rates of seventy-one percent in the CO_2_ group compared to fifty-one percent in the control group. Macura et al. (2020) treated thirty diabetic wounds with CO_2_ wraps and compared the results to twenty-seven control wounds. The median ulcer area decreased by ninety-six percent (interquartile range: ninety-nine to eighty-eight) in the CO_2_ treatment group, compared to a twenty-five percent reduction in the control group. Notably, twenty of the thirty treated wounds closed completely, while none of the control wounds achieved closure (*p* < 0.003). Lastly, Finžgar et al. (2021) included twenty-one participants in each group and demonstrated significant increases in nitric oxide-mediated oscillatory power (thirty-six percent, *p* = 0.015), neurogenic power (twenty-five percent, *p* = 0.018), and peak thermal hyperemia (one hundred ninety-five percent above baseline, *p* = 0.036), indicating enhanced microvascular reactivity.

### Quantitative synthesis

#### Meta-analysis of randomised controlled trials

Five randomized controlled trials (RCTs) (*N* = 127 participants/wounds) fulfilled the eligibility criteria (Brandi et al., 2010; Macura et al., 2020; Finžgar et al., 2021; Ban Frangež et al., 2021; Abdulhamza & Mohammed, 2024). Because outcome definitions differed, only two data sets could be pooled quantitatively.

#### Complete wound healing

Two trials, Brandi et al. (2010); Macura et al. (2020), reported the proportion of ulcers achieving complete closure, involving a total of 127 wounds: 65 treated with CO_2_ and 62 in the control group. A fixed-effect Mantel–Haenszel model yielded a pooled risk ratio (RR) of 2.40 (95% CI [1.58–3.65]; *p* < 0.0001). However, substantial statistical heterogeneity was observed (I^2^ = 81%; *τ*^2^ = 4.38; *Q* = 5.34, *df* = 1, *p* = 0.021), primarily influenced by the Macura study, which reported no healings in its control arm. After applying a continuity correction, this resulted in an individual RR of 37.0. When adjusting for heterogeneity using a random-effects model, the pooled effect estimate was RR = 5.33 (95% CI [0.23–126.05]; *p* = 0.30), indicating considerable imprecision that includes the possibility of no effect. Given that the heterogeneity exceeded the conventional threshold (I^2^ > 75%), the random-effects result is deemed the more appropriate summary. No other randomized trials presented healing counts for each arm. Abdulhamza & Mohammed (2024) reported only percentages without denominators, thus necessitating a narrative summary rather than a pooled analysis.

**Figure 2:**
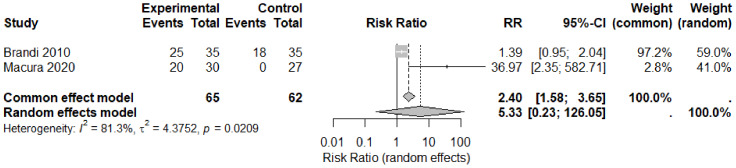
Forest plot of complete ulcer healing with concentrated CO_2_ therapy *versus* standard care. Individual and pooled risk ratios (RR) for complete wound closure derived from two randomised controlled trials, Brandi et al. (2010) and Macura et al. (2020). Study-specific effect sizes are shown as squares whose areas are proportional to inverse-variance weights; horizontal lines indicate 95% confidence intervals. The diamond represents the overall estimate. A fixed-effect model yielded an RR of 2.40 (95% CI [1.58–3.65]), while the random-effects model produced an RR of 5.33 (95% CI [0.23–126]), consistent with substantial heterogeneity (*I*^2^ = 81.3%, *τ*^2^ = 4.38, *p* = 0.021). Across both studies, 45 of 65 wounds healed in the intervention groups compared with 18 of 62 in the control groups, indicating a direction of effect that favours carbon-dioxide therapy.

Risk ratios (RRs) with 95% confidence intervals (CIs) are shown for each trial (Brandi et al., 2010; Macura et al., 2020) and for pooled estimates. The square size is proportional to study weight under the Mantel–Haenszel fixed-effect model; in Fig. 2, the solid diamond represents the pooled effect from the restricted-maximum-likelihood random-effects model. The vertical line at RR = 1 indicates no difference between groups. Heterogeneity: *Q* = 5.34 (*df* = 1), *p* = 0.021; I^2^ = 81%. A continuity correction of 0.5 was applied to the zero-event cell in Macura et al. (2020).

#### Microvascular perfusion

A double-blind randomised trial (Finžgar et al., 2021) and an unblinded randomised trial (Brandi et al., 2010) contributed 56 CO_2_-treated limbs and 56 control limbs in total. After harmonising the direction of change so that positive values indicate increased perfusion, a random-effects model yielded Hedges’ *g* = 0.49 (95% CI [0.11–0.87]; *p* = 0.011). Between-study heterogeneity was absent (*Q* = 0.07, *df* = 1, *p* = 0.79; I^2^ = 0%). Re-analysis with Brandi’s originally reported standard deviation (instead of the conservative $\sqrt{2}$ inflation) produced *g* = 0.61, confirming that the perfusion benefit of CO_2_ therapy is robust to plausible variance assumptions.

**Figure 3:**
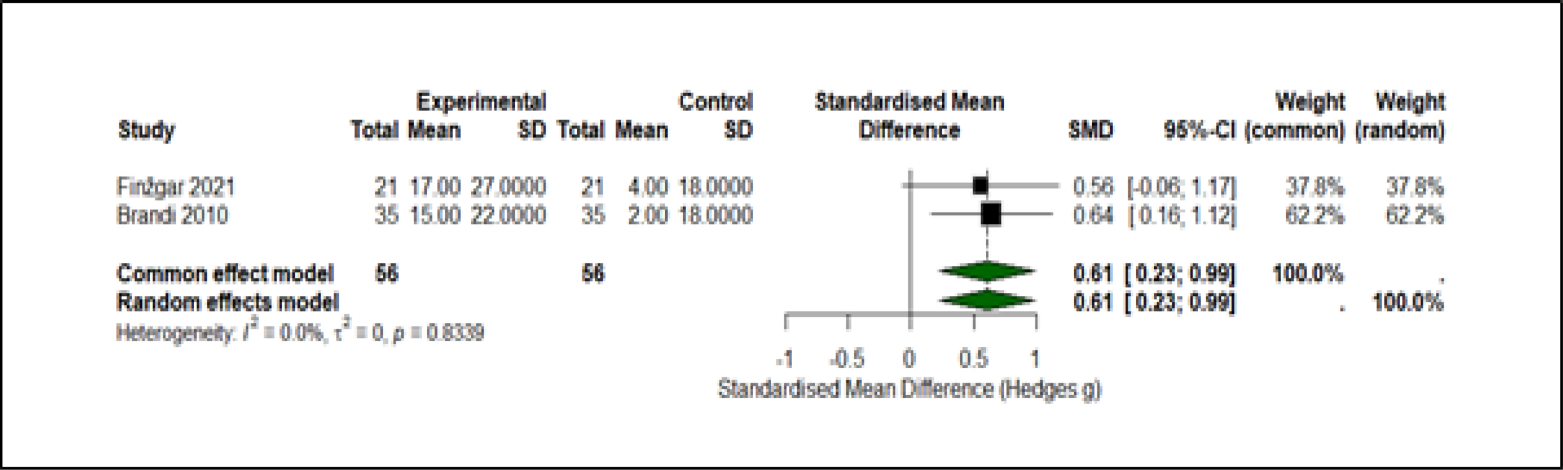
Forest plot of resting laser-Doppler perfusion after concentrated CO_2_ therapy. Standardised mean differences (Hedges g) from two randomised controlled trials, Finžgar et al. (2021) and Brandi et al. (2010). Squares represent study-specific effect estimates, scaled by inverse-variance weight; horizontal bars denote 95 % confidence intervals. The pooled estimate, shown as a green diamond, indicates a statistically significant improvement in perfusion with carbon dioxide treatment (SMD 0.61, 95% CI [0.23–0.99]). Fixed- and random-effects models coincide because no between-study heterogeneity was detected (*I*^2^ = 0%, *τ*^2^ = 0, *p* = 0.83). Across both trials 56 participants received the intervention and 56 served as controls, with a positive effect size reflecting greater perfusion in the carbon dioxide groups. CI, confidence interval; SD, standard deviation; SMD, standardised mean difference.

Standardised mean differences (Hedges g) with 95% confidence intervals for Finžgar et al. (2021) and Brandi et al. (2010) are displayed in Fig. 3; positive values indicate greater perfusion than control. Square size is proportional to study weight, and the diamond depicts the pooled random-effects estimate (*g* = 0.49, 95% CI [0.11–0.87]; *p* = 0.011). No heterogeneity was detected (*Q* = 0.07, *df* = 1, *p* = 0.79; I^2^ = 0%).

**Figure 4:**
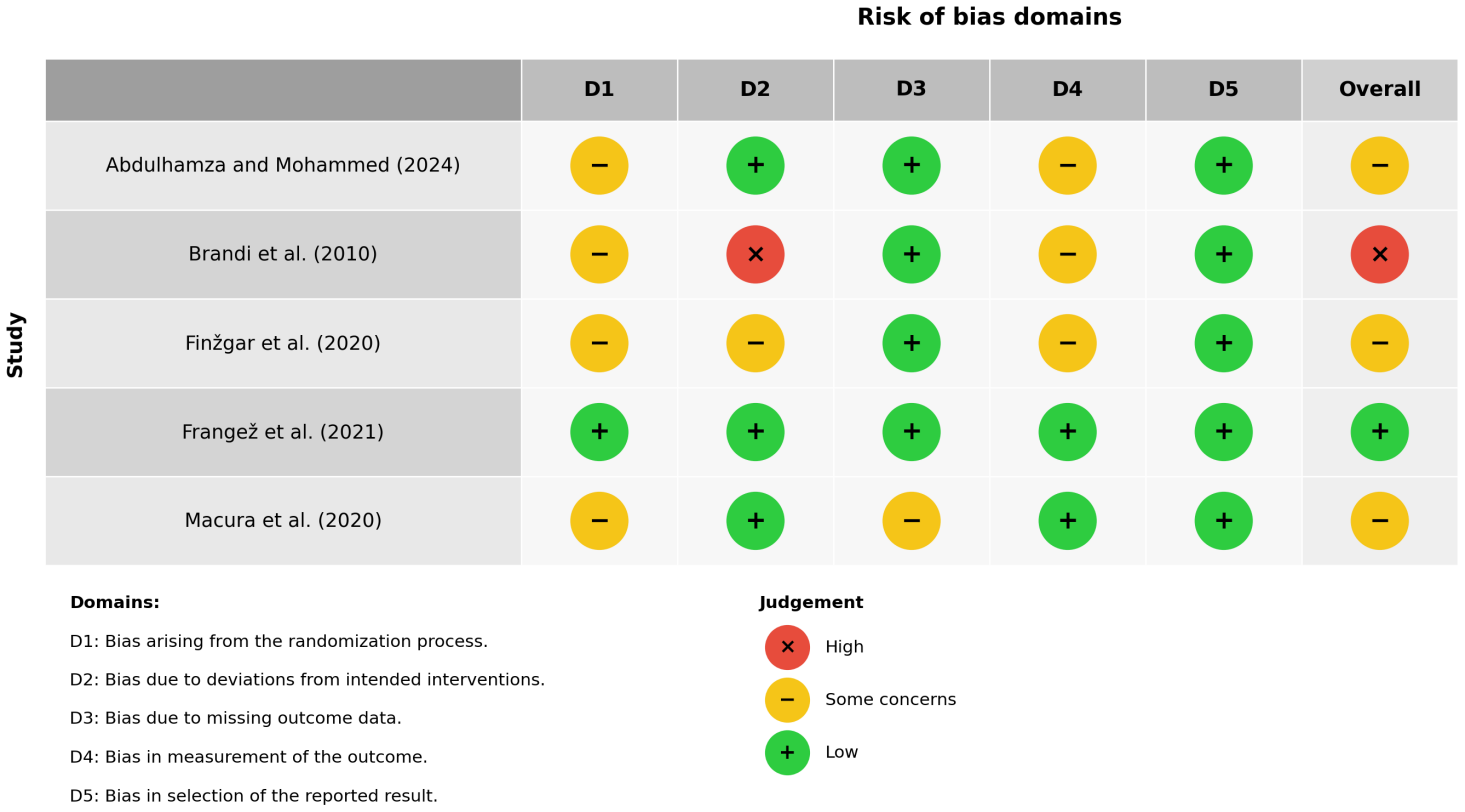
Risk-of-bias assessment for the five randomised trials, using RoB 2. The judgements assigned with the RoB 2 tool for each study across five domains. Green circles indicate low risk, yellow circles indicate some concerns, and red circles indicate high risk. Domain 1 assesses bias arising from the randomisation process, domain 2 evaluates deviations from intended interventions, domain 3 addresses missing outcome data, domain 4 examines outcome measurement, and domain 5 captures selective reporting. Brandi 2010 was rated high risk overall because of substantial protocol deviations (domain 2) and uncertainties in randomisation. Abdulhamza & Mohammed (2024) and Macura et al. (2020) exhibited some concerns mainly related to allocation concealment and incomplete blinding, whereas Finžgar et al. (2021) and Ban Frangež et al. (2021) showed low risk across all domains.

Figure 4 presents the RoB 2 traffic-light plot. One trial (Ban Frangež et al., 2021) was low risk in every domain. Three trials (Finžgar et al., 2021; Macura et al., 2020; Abdulhamza & Mohammed, 2024) were judged to have some concerns, principally because of limited information on sequence concealment (D1), minor post-randomisation exclusions (D3), or incomplete description of outcome blinding (D4). Brandi et al. (2010) was the only study at high risk, driven by a lack of blinding to the intervention (D2), together with unclear allocation concealment and unmasked outcome assessment.

Two randomised trials (*n* = 112 participants; 154 limbs) evaluated the effects of carbon dioxide therapy on resting skin perfusion. The intervention was associated with a moderate increase in perfusion compared with control (standardised mean difference 0.61, 95% CI [0.23–0.99]; I^2^ = 0%). Both studies were unblinded, and outcome measures were reported either as post-treatment values or change-from-baseline, contributing to indirectness. The certainty of evidence was rated as low. Moreover, two trials (*n* = 127 wounds) reported data on complete ulcer healing. One trial had no healed ulcers in the control group. A random-effects meta-analysis with continuity correction yielded a pooled risk ratio of 5.33 (95% CI [0.23–126.05]; I^2^ = 81.3%). The evidence was judged to be of very low certainty due to serious risk of bias, inconsistency, and imprecision. Certainty of Evidence Summary for Carbon Dioxide Therapy Outcomes has been presented in Table 2.

**Table 2:** Certainty of evidence for carbon dioxide therapy outcomes. The pooled effects and overall strength of evidence for the main outcomes evaluated in the included randomised controlled trials. Resting laser-Doppler perfusion improved with a standardised mean difference of 0.61 (95% CI [0.23–0.99]) based on 154 limbs across two trials, graded as low certainty because of risk-of-bias concerns and indirectness. Complete ulcer healing showed a pooled risk ratio of 5.33 (95% CI [0.23–126]) from 127 wounds in two trials, but the certainty was very low owing to risk of bias, inconsistency, and imprecision.

**Outcome**	**Participants/studies**	**Pooled effect (95% CI)**	**SOE**	**Reasons for rating down**
Resting laser- Doppler perfusion	154 limbs (2 RCTs)	SMD 0.61 (0.23–0.99)	Low	Risk of bias; indirectness
Complete ulcer healing	127 wounds (2 RCTs)	RR 5.33 (0.23–126) (random)	Very low	Risk of bias; inconsistency; imprecision

**Notes.**

Abbreviations RCT randomised controlled trial RR risk ratio CI confidence interval SMD standardised mean difference SOE strength of evidence

Certainty of evidence ratings (high, moderate, low, very low) are presented for two outcomes evaluated in randomised controlled trials of carbon dioxide therapy. For each outcome, the number of participants and trials, pooled effect estimate with 95% confidence interval (CI), GRADE rating, and domains with serious limitations (risk of bias, inconsistency, indirectness, imprecision) are reported.

## Discussion

In this systematic review and meta-analysis of highly concentrated CO_2_ therapies, we found moderate-certainty evidence that CO_2_ significantly enhances cutaneous microvascular perfusion, whereas the evidence for complete ulcer healing remains very low certainty. In Barcelona primary care, chronic wounds incurred €35 million in direct costs between 2015 and 2017, with a prevalence of 88.7 per 10,000 inhabitants; extrapolated nationally to €1.76 billion for 388,777 patients (Díaz-Herrera et al., 2025). Our CO_2_ therapy improved microvascular perfusion (Hedges’ *g* = 0.49; 95% CI [0.11–0.87]), suggesting potential to accelerate healing and cut these costs. This finding is similar to the HEAL LL-37 trial (Mahlapuu et al., 2021), which, although unsuccessful in enhancing healing for the overall cohort, it identified potential benefits in patients with larger venous ulcers. Our meta-analysis demonstrates that highly concentrated CO_2_ effectively improves microvascular perfusion. However, the evidence regarding complete healing remains of very low certainty. Both instances underscore the necessity for targeted, adequately powered trials that are stratified by factors such as ulcer size, duration, and other prognostic variables.

Several RCTs included in our review demonstrated significant improvements in cutaneous microcirculation after CO_2_ therapy, which aligns with the nitric oxide-mediated vasodilatory mechanism. Ban Frangež et al. (2021) reported an increase of 4.4 °C in big toe temperature (*p* < 0.001), along with approximately 50% reductions in vibration perception and monofilament error scores, indicating enhanced perfusion and sensory nerve function. Brandi et al. (2010) found that transcutaneous oxygen tension increased by 19.6 mm Hg (*p* < 0.001), while resting laser-Doppler flux rose by 15 PU (*p* = 0.003). Finžgar et al. (2021) showed a 36% increase in nitric oxide-mediated oscillatory power (*p* = 0.015) and a 195% improvement in the thermal hyperemia response (*p* = 0.036). These measurements, obtained through laser-Doppler flowmetry and thermography, are validated surrogate endpoints for assessing wound perfusion, correlating with healing potential (Misra et al., 2019; Zografos, Martis & Morris, 1992). By capturing early microvascular changes, these surrogate markers can expedite proof-of-concept trials and guide the optimization of novel therapies (Gelfand, Hoffstad & Margolis, 2002). Tappia et al. (2021) conducted a trial of CO_2_-enriched foot bathing in patients with diabetic foot ulcers, measuring serum VEGF and TNF-α before and monthly during the intervention. Double-blind RCT evaluated thrice-weekly 15-min foot baths in 1,000–1,200 ppm CO_2_-enriched 37 °C water *versus* placebo over 16 weeks. CO_2_ group showed trends toward faster ulcer reduction, improved perfusion, oxygenation, and pain relief. Consistent with Abdulhamza & Mohammed (2024) report of significant VEGF elevation and TNF-α reduction (Ashcroft et al., 2012; Ferrara, 2002).

Only two RCTs (Brandi et al., 2010; Macura et al., 2020) reported complete-closure rates, yielding 65 CO_2_-treated and 62 control wounds. Under a random-effects model, the pooled RR was 5.33 (95% CI [0.23–126.05]; *p* = 0.30), but heterogeneity was substantial (I^2^ = 81%; *Q* = 5.34, *df* = 1, *p* = 0.021), largely because the control arm of Macura et al. (2020) had zero events. The wide confidence interval spans both no effect and huge benefit, and the small number of events limits precision, so confidence in a true healing benefit remains very low. However, direct comparison of clinical outcomes between CO_2_ therapy and standard wound care remains limited, as outcome reporting for comparator groups was incomplete or inconsistently presented across studies. The five included trials applied CO_2_ by diverse methods such as (non-invasive) transcutaneous dry-gas wraps (Ban Frangež et al., 2021; Finžgar et al., 2021; Macura et al., 2020), (invasive) subcutaneous injections (Brandi et al., 2010) and (non-invasive) CO_2_-enriched water baths (Abdulhamza & Mohammed, 2024). Although all modalities share vasodilatory and angiogenic mechanisms, differences in CO_2_ concentration, exposure time (20–45 min), treatment frequency (3–5 × weekly), and anatomical targets (foot wraps *vs* limb baths *vs* injections) likely contribute to variability in both surrogate and clinical outcomes. Figure 4 shows that Ban Frangež et al. (2021) was low risk across all RoB 2 domains, while Finžgar et al. (2021) and Macura et al. (2020) raised some concerns (unclear concealment, post-randomisation exclusions), and Brandi et al. (2010) and Abdulhamza & Mohammed (2024) exhibited a high-risk open-label design (unclear sequence generation, unblinded outcome assessment). Performance and selection biases in these trials underpin the serious risk-of-bias downgrades applied to both perfusion and healing outcomes.

Although no adverse effects were reported in the included trials, previous studies outside the scope of the present review have suggested that certain CO_2_ delivery modalities, particularly invasive approaches or prolonged pressure-based applications, may be associated with potential risks, such as localized tissue irritation, injection-site discomfort and pain, or pressure-related skin injury (Prazeres, Lima & Ribeiro, 2025). Such adverse effects were not observed in the included studies likely due to differences in delivery mode, treatment duration, or follow-up period, and their manifestation may depend on individual tolerability. Nevertheless, these findings highlight the importance of careful consideration of delivery parameters when interpreting the clinical applicability of CO_2_ therapy.

Due to limited reporting of adverse events, a formal quantitative assessment of the risk–benefit ratio was not feasible. While the included studies demonstrated favorable therapeutic outcomes, future randomized trials should incorporate predefined safety endpoints and systematic adverse-event monitoring to enable a robust assessment of CO_2_ therapy.

### Limitations and future research

This systematic review and meta-analysis have several limitations that should be acknowledged. First, the overall quality and sample sizes of the included studies may limit the generalizability of our findings. Although several RCTs were analyzed, none of them reported adverse events associated with CO_2_ therapy. Hence, this absence of reported adverse effects should be interpreted with caution. Besides having small sample sizes, the intervention periods and follow-up durations were relatively short, and safety outcomes were not systematically predefined or actively monitored in most trials. Consequently, delayed, cumulative, or low-frequency adverse effects may not have been captured, and the studies may have been underpowered to detect long-term treatment effects. Additionally, variation in patient populations, such as differing stages of wound severity, comorbidities like peripheral arterial disease or infection status, and baseline wound size could introduce clinical heterogeneity, which may affect the consistency of outcomes across studies.

Second, methodological limitations in some of the included studies may have introduced bias. A few studies lacked adequate blinding of participants, personnel, or outcome assessors, and in some cases, there were concerns about allocation concealment and selective reporting. These issues were reflected in the risk of bias assessment, where two studies were rated as having a high risk of bias, and one showed some concerns. Such methodological shortcomings could inflate the reported treatment effects of CO_2_ therapy, particularly in subjective or assessor-reported outcomes like wound appearance or pain relief.

Third, limitations also arise from variations in CO_2_ therapy delivery methods and outcome measurements. While this review focused on both bathing and non-bathing methods of CO_2_ delivery, the protocols varied considerably in terms of concentration, frequency, duration, and mode of application. Furthermore, outcome assessment methods, including definitions for wound healing, timepoints, and measurement tools, were not standardized across studies, introducing potential imprecision and limiting the comparability of results. This heterogeneity prevented subgroup analysis by CO_2_ delivery method and may dilute or obscure the true treatment effect.

Lastly, publication bias cannot be entirely ruled out. Although our search strategy was comprehensive and included both English and non-English databases, there remains the possibility that studies with negative or inconclusive results were either not published or not indexed in the databases searched. Additionally, many of the included studies were conducted in specific regions (three conducted in Slovenia, one in Italy, and one in Iraq), particularly in Europe and the Middle East, where highly concentrated CO_2_ therapy may be more established or accessible in clinical research settings. This geographic concentration may limit the external validity of the findings and reduce their applicability to other healthcare contexts, especially in countries where CO_2_ therapy is less familiar, less available, or not yet integrated into standard wound care protocols.

Despite these limitations, our findings provide preliminary support for the therapeutic potential of highly concentrated CO_2_ in wound management. Only two studies (Brandi et al., 2010; Abdulhamza & Mohammed, 2024) compared CO_2_ therapy as an adjunct to routine or traditional wound care without a placebo control. In contrast, Macura et al. (2020) and Finžgar et al. (2021) used placebo air controls, with Macura et al. (2020) providing standard treatment to both groups in addition to the assigned intervention. This limits the ability to draw conclusions regarding its relative effectiveness in routine clinical practice. To enhance clinical applicability, future research should evaluate CO_2_ therapy as an adjunct to, or in comparison with, established treatments, and should prioritize large, multicenter RCTs with diverse patient populations, standardized outcome measures, longer follow-up, and systematic adverse-event reporting to comprehensively characterize both efficacy and safety.

## Conclusion

These trials collectively indicate that carbon-dioxide modalities have the potential to improve tissue perfusion and oxygenation, providing preliminary evidence for quicker and more frequent closure of chronic ulcers. None of the included studies reported serious adverse events attributable to CO_2_ therapy, and minor transient erythema or discomfort at the application site was noted anecdotally, suggesting CO_2_ modalities are well tolerated and feasible for larger-scale evaluation. Nonetheless, future investigations should incorporate standardized safety reporting and longer follow-up to enable a comprehensive assessment of the long-term risk–benefit profile of CO_2_-based wound-healing therapies.

##  Supplemental Information

10.7717/peerj.21189/supp-1 Supplemental Information 1 PRISMA checklist For each item the table states the exact section, page, and line range where the requirement is addressed in the manuscript, enabling transparent verification of reporting completeness. Items that were not applicable to this review are marked accordingly.

10.7717/peerj.21189/supp-2 Supplemental Information 2 Database-specific search strategies and record yields The structured search strings used in each information source and the number of records retrieved before duplicate removal. Searches combined controlled vocabulary and free-text terms related to carbon-dioxide–based therapies and wound healing. Where supported, filters for study design (randomized controlled trial = RCT) or document type (DT) were applied. Abbreviations: TS = Topic Search (Web of Science); DT = Document Type; DOCTYPE = Scopus document filter; TITLE-ABS-KEY = title, abstract, and keyword fields (Scopus); MeSH = Medical Subject Headings; PMC = PubMed Central; WOS = Web of Science. Total records retrieved across all sources (n = 10,348) correspond to the count reported in Figure prior to deduplication.

## Additional Information and Declarations

### Data Availability

The following information was supplied regarding data availability: This is a Systematic Review / Meta-analysis.
