# Supplementary material for: Therapeutic use of highly concentrated CO2 for wound healing: bathing and non-bathing modalities in a systematic review and meta-analysis
Source: PeerJ. 2026 May 5;14:e21189. doi: 10.7717/peerj.21189 (PMC13155233; doi:10.7717/peerj.21189)
Supplement: Supplemental Information 2 — The structured search strings used in each information source and the number of records retrieved before duplicate removal. Searches combined controlled vocabulary and free-text terms related to carbon-dioxide–based therapies and wound healing. Where supported, filters for study design (randomized controlled trial = RCT) or document type (DT) were applied. Abbreviations: TS = Topic Search (Web of Science); DT = Document Type; DOCTYPE = Scopus document filter; TITLE-ABS-KEY = title, abstract, and keyword fields (Scopus); MeSH = Medical Subject Headings; PMC = PubMed Central; WOS = Web of Science. Total records retrieved across all sources (n = 10,348) correspond to the count reported in Figure prior to deduplication. [file peerj-14-21189-s002.docx]

**Supplementary Table 1**: A summary of the search strategies used across each database.

| **Database** | **Search Strategy** | **Studies Identified** |
| --- | --- | --- |
| **PubMED** | ((((((((carbon dioxide bath[Title/Abstract]) OR (CO2 bathing[Title/Abstract])) OR (transcutaneous carbon dioxide[Title/Abstract])) OR (CO2 therapy[Title/Abstract])) AND (wound healing[Title/Abstract])) OR (chronic wound[Title/Abstract])) OR (diabetic foot ulcer[Title/Abstract])) OR (skin ulcer[Title/Abstract])) AND (humans[MeSH Terms]) | 5,753 |
|  | ((((carbon dioxide bath[Title/Abstract]) OR (CO2 bathing[Title/Abstract])) OR (transcutaneous CO2[Title/Abstract])) AND (wound healing[Title/Abstract])) AND (randomized controlled trial[Publication Type]) | 1 |
|  | (((((((carbon dioxide therapy[Title/Abstract]) OR (CO2 hydrotherapy[Title/Abstract])) OR (transdermal CO2[Title/Abstract])) OR (CO2-rich water[Title/Abstract])) AND (wound healing[Title/Abstract])) OR (skin regeneration[Title/Abstract])) OR (diabetic wound[Title/Abstract])) AND (human[MeSH Terms]) | 2,400 |
| **PMC** | ("carbon dioxide bath" OR "CO2 bathing" OR "transcutaneous CO2" OR "carbon dioxide therapy") AND ("wound healing" OR "chronic wound" OR "diabetic foot ulcer" OR "skin ulcer") | 50 |
|  | ("transdermal CO2" OR "CO2 hydrotherapy" OR "CO2-rich water" OR "topical carbon dioxide")  AND  ("wound healing" OR "diabetic wound" OR "skin regeneration") | 5 |
|  | ("carbon dioxide bath" OR "CO2 therapy" OR "transcutaneous CO2")  AND  ("wound healing" OR "skin ulcer")  AND  ("randomized trial" OR "clinical trial" OR "RCT" OR "intervention study") | 24 |
| **WOS** | TS=("carbon dioxide bath" OR "CO2 bathing" OR "transcutaneous CO2" OR "CO2 therapy")  AND  TS=("wound healing" OR "chronic wound" OR "diabetic foot ulcer" OR "skin ulcer")  AND  DT=(Article OR Clinical Trial) | 6 |
|  | TS=("CO2 hydrotherapy" OR "transdermal CO2" OR "CO2-rich water" OR "topical carbon dioxide")  AND  TS=("wound healing" OR "ulcer" OR "skin regeneration")  AND  DT=(Article OR Clinical Trial) | 4 |
|  | TS=("carbon dioxide bath" OR "CO2 therapy" OR "transcutaneous CO2")  AND  TS=("wound healing" OR "diabetic wound" OR "pressure ulcer")  AND  TS=("randomized trial" OR "clinical trial" OR "intervention study")  AND  DT=(Article) | 1 |
| **Scopus** | TITLE-ABS-KEY("carbon dioxide bath" OR "CO2 bathing" OR "transcutaneous CO2" OR "CO2 therapy")  AND  TITLE-ABS-KEY("wound healing" OR "chronic wound" OR "diabetic foot ulcer" OR "skin ulcer")  AND  DOCTYPE(ar) AND  LANGUAGE(english) | 11 |
|  | TITLE-ABS-KEY("transdermal CO2" OR "CO2 hydrotherapy" OR "CO2-rich water" OR "topical carbon dioxide")  AND  TITLE-ABS-KEY("diabetic foot ulcer" OR "pressure ulcer" OR "skin regeneration" OR "non-healing wound")  AND  DOCTYPE(ar)  AND  LANGUAGE(english) | 1 |
|  | TITLE-ABS-KEY("carbon dioxide bath" OR "CO2 therapy" OR "transcutaneous CO2")  AND  TITLE-ABS-KEY("wound healing" OR "ulcer")  AND  TITLE-ABS-KEY("randomized controlled trial" OR "clinical trial" OR "intervention study" OR "RCT")  AND  DOCTYPE(ar)  AND  LANGUAGE(english) | 2 |
| **Clinical Trial** | "carbon dioxide bath" OR "CO2 therapy" OR "transcutaneous carbon dioxide" AND "wound healing" OR "chronic wound" OR "diabetic ulcer" OR "skin ulcer" | 2045 |
|  | ("carbon dioxide therapy" OR "CO2-enriched water" OR "transcutaneous carbon dioxide")  AND  ("wound" OR "ulcer") | 18 |
| **Google**  **Scholar** | "carbon dioxide bath" "wound healing" | 14 |
|  | "CO2 therapy" "diabetic ulcer" | 4 |
|  | "transcutaneous carbon dioxide" "skin ulcer" | 4 |
|  | "topical carbon dioxide" "pressure ulcer" | 1 |
|  | "transdermal CO2" "tissue regeneration" | 2 |
|  | "CO2-enriched water" "skin healing" | 2 |
| **Total** | | 10,348 |
